# Vapor-Phase Halogenation of Hydrogen-Terminated Silicon(100) Using *N*-Halogen-succinimides

**DOI:** 10.1021/acsami.3c13269

**Published:** 2023-11-15

**Authors:** Patrick R. Raffaelle, George T. Wang, Alexander A. Shestopalov

**Affiliations:** † Department of Chemical Engineering, Hajim School of Engineering and Applied Sciences, University of Rochester, Rochester, New York 14627, United States; ‡ Sandia National Laboratories, Albuquerque, New Mexico 87185, United States

**Keywords:** silicon halogenation, area-selective atomic layer deposition, surface functionalization, atomic resists, vapor-phase deposition

## Abstract

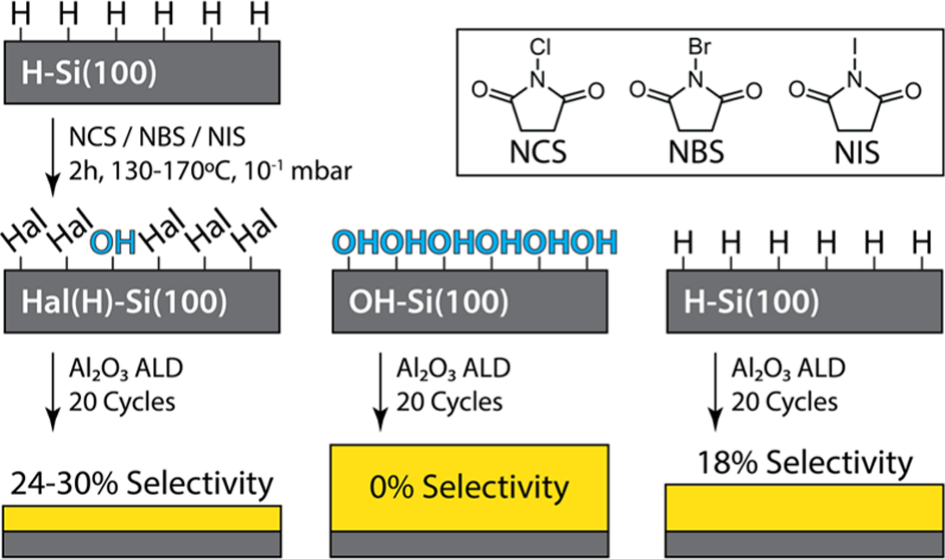

The focus of this study was to demonstrate the vapor-phase halogenation of Si(100) and subsequently evaluate the inhibiting ability of the halogenated surfaces toward atomic layer deposition (ALD) of aluminum oxide (Al_2_O_3_). Hydrogen-terminated silicon ⟨100⟩ (H–Si(100)) was halogenated using *N*-chlorosuccinimide (*N*CS), *N*-bromosuccinimide (NBS), and *N*-iodosuccinimide (NIS) in a vacuum-based chemical process. The composition and physical properties of the prepared monolayers were analyzed by using X-ray photoelectron spectroscopy (XPS) and contact angle (CA) goniometry. These measurements confirmed that all three reagents were more effective in halogenating H–Si(100) over OH–Si(100) in the vapor phase. The stability of the modified surfaces in air was also tested, with the chlorinated surface showing the greatest resistance to monolayer degradation and silicon oxide (SiO_2_) generation within the first 24 h of exposure to air. XPS and atomic force microscopy (AFM) measurements showed that the succinimide-derived Hal-Si(100) surfaces exhibited blocking ability superior to that of H–Si(100), a commonly used ALD resist. This halogenation method provides a dry chemistry alternative for creating halogen-based ALD resists on Si(100) in near-ambient environments.

## Introduction

Area-selective atomic layer deposition (AS-ALD) is a key technology for enabling atomically precise, self-aligned bottom-up manufacturing of thin-film electronic,^1,2^ photonic,^3^ and quantum devices.^4^ This method is often suggested as a higher-resolution replacement for current top-down micromachining techniques that can further advance the development of three-dimensional (3D) integrated circuits.^5^ AS-ALD relies on patterned interfaces of growth and nongrowth surfaces (GS and NGS), which sequentially promote and block ALD reactions on the corresponding domains.^6−9^ Growth selectivity is typically achieved by patterning ALD resists that block deposition on homogeneous growth-promoting interfaces.

Typical AS-ALD resists include polymeric^10−14^ and inorganic^15−19^ thin films. They are often deposited via traditional stencil lithography or photolithography, negating the potential high-resolution advantages of AS-ALD. Alternative resist materials include self-assembled monolayers (SAMs) which possess advantages over more traditional resists.^20−24^ However, these SAM resists still consist of relatively large molecules (up to 2 nm in length). SAM resists are also predominantly deposited via solution chemistry incompatible with in situ ALD, and their properties are altered near surface defects making their use on nonideal interfaces problematic.

Considering the limitations of SAM resists, recent efforts in AS-ALD have been directed toward the development of atomic resists that can promote or block ALD reactions by changing the surface termination of the deposition substrate. These studies are focused on developing chemoselective atomic resists that can withstand multiple ALD cycles without losing their growth selectivity (i.e., the number of cycles before a resist loses its blocking ability).^25−28^

Past studies have examined the effectiveness of ALD resists which focus on functionalizing Si(100) interfaces.^29,30^ In the (100) orientation, the Si/SiO_2_ interface state density is generally lower than those in the (110) and (111) planes, resulting in a lower density of dangling bonds, higher carrier mobility, and better drive current for Si-based electronics. Currently, hydrogen-terminated silicon (H–Si(100)) is employed as an effective NGS when paired with hydroxylated silicon (OH–Si(100)) which acts as the GS. This basic complementary resist system is valued for its uniform composition and good growth/etch selectivity.^8,31^ However, H–Si(100) is reactive and unstable in air, limiting its use in ultrahigh vacuum (UHV) environments and hindering its application in commercial systems for bottom-up processing. Therefore, alternatives to H–Si(100) that maintain its attractive physical and chemical properties, inhibit partial oxidation, and improve the ALD growth selectivity with OH–Si(100) are needed.

Halogenated Si(100) (Hal-Si(100)) is a promising candidate for fabricating semiconductor interfaces used in electronics,^32^ nanotechnology,^33,34^ and biosensing.^35,36^ It is a more stable alternative to H–Si(100) in air and can better maintain the required difference in chemical reactivity on a surface for AS-ALD due to the bulkier structure of the attached halogen species, which effectively shield the underlying NGS from ALD chemistry.^37^ Different halogen precursors can be used to passivate Si(100), potentially enabling versatility in inhibiting different types of ALD chemistries. For example, the reaction of 1-octadecanethiol is more favorable on a Cl-terminated Si(100) surface (Cl–Si(100)) than on a Br-terminated surface (Br–Si(100)).^38^ Cl–Si(100) also lowers the processing temperature for depositing stable NH_2_ groups on silicon surfaces using gas-phase ammonia. Furthermore, halogen atoms have demonstrated great potential as possible passivation species for pattern preservation on H-passivated materials under UHV.^39^

Most research on halogenation of crystalline silicon has focused on the Si(111) interface,^32,34−36^ but the structural differences between Si(111) and Si(100) result in different reactivities.^40,41^ Thus, there is a need to investigate halogen formation on Si(100) more extensively, as it is a more relevant material in semiconductor manufacturing.^42^ The current standard for producing Hal-Si(100) involves generating a halogen flux from a solid-state, electrochemical cell in UHV,^43−46^ which is slow and not directly compatible with common commercial deposition systems. Others have employed chlorine gas (Cl_2_) under low-pressure conditions to chlorinate Si(111);^47,48^ however, due to the toxicity of Cl_2_ gas and its problematic incorporation into most vacuum deposition systems, there is a need to find other halogenation molecules that are safer to handle. Some have developed Si(100) halogenation methods using wet chemistry in near-ambient conditions,^34,47,49−51^ but these often require prolonged refluxing and generate byproducts that accumulate on the Si(100) surface. Vapor-phase halogenation in mild vacuum is more compatible with other low-vacuum processes such as AS-ALD, but few studies outside of UHV have focused on vapor-phase halogenation of H–Si(100). Nonetheless, H-terminated silicon quantum dots (H-SiQDs) were halogenated using chlorine gas (Cl_2_) and *N*-halogen-succinimides^38,52−56^ but both methods have resulted in SiQD oxidation and only partial halogenation. Thus, a more efficient vapor-phase halogenation method for Si(100) is needed.

This study reports the use of *N*-halogen-succinimide molecules as vapor-phase halogenation reagents for crystalline H–Si(100) under near-ambient conditions. *N*-Halogen-succinimide molecules are often used as a precursor of molecular halogens in radical-type reactions.^34,52,57,58^ The reaction likely follows a surface-mediated radical initiator mechanism where the terminal H atoms on a H–Si(100) surface are replaced by Cl, Br, or I atoms. This reaction is likely to proceed through the formation of silicon radicals and molecular halogens, analogous to the allylic or benzylic halogenation of alkanes or alkylbenzenes in the solution phase.^59^

The halogen precursors used in this study were *N*-chlorosuccinimide (NCS), *N*-bromosuccinimide (NBS), and *N*-iodosuccinimide (NIS), which are all commonly used as halogenating and oxidizing agents in organic synthesis and can be handled in air.^35,52,60,61^ The resulting monolayers were characterized by using X-ray photoelectron spectroscopy (XPS) and contact angle goniometry. Calculations of surface coverage suggest that the succinimide halogenation achieves a high level of coverage, albeit not complete. The reported halogenation reaction has been shown to have high chemoselectivity and can be used to selectively halogenate H–Si(100) in the presence of hydroxyl-terminated surface sites. The stability of the Hal-Si(100) interfaces in air was investigated using XPS to monitor the concentration of oxide and halogen species on Si(100) over a 72 h period. The results showed that the halogenated substrates degraded at a slower rate than that of H–Si(100). When compared to other vapor-phase halogenation techniques (i.e., halogen flux from an electrochemical cell in UHV, surface exposure to Cl_2_ gas), *N*-Hal-succinimides do not require high vacuum environments, are safer to handle, and less corrosive to the deposition instrumentation. The described vapor-phase halogenation conditions are also comparable to the conditions of existing ALD chemistries and can potentially be undertaken inside standard ALD systems. They could be used as an alternative to H resists on Si(100) in self-aligned AS-ALD. Here, we studied the selectivity of the ALD of alumina (Al_2_O_3_) on Hal-Si(100) and on H–Si(100). XPS and angle-resolved XPS (ARXPS) data showed that the halogenated monolayers were better at inhibiting the ALD chemistry than H–Si(100). We believe that the development of highly selective and stable ALD resists that can be deposited under mild vapor-phase conditions would have numerous applications in bottom-up semiconductor manufacturing. However, more research is needed to understand the mechanism of defect formation during and after the reaction to further improve the coverage and stability of the halogen resists.

## Materials and Methods

All reagents and solvents were used as received without further purification. Solvents were purchased from Sigma-Aldrich and filtered through a 0.2 μm filter before use. Light-sensitive molecules NCS, NBS, and NIS all with 99% purity were purchased from Sigma-Aldrich and stored in dark environments. Their application was carried out under yellow light. P-doped ⟨100⟩ silicon wafers were purchased from University Wafer, Boston, Massachusetts. XPS spectra were recorded on a Kratos Axis Ultra XPS spectrometer equipped with a mono-Al X-ray source at 200 W power and a pressure of 3.0 × 10^–8^ mbar. Survey scans were obtained between 0 and 1200 eV with a step size of 1 eV, a dwell time of 200 ms, and a pass energy of 140 eV averaged over 2 scans. Core-level region scans were obtained at the corresponding binding energy ranges with a step size of 0.1 eV, an average dwell time of 260 ms, and a passing energy of 20 eV averaged over 10 scans. Data was processed using CasaXPS software and instrument-specific atomic sensitivity factors. All C 1s peaks were calibrated to 284 eV, and this same binding energy shift was applied to all other spectra besides Si 2p to account for adventitious carbon contamination. Separately, the bulk Si signal in the Si 2p spectra was calibrated to 99 eV for better quantitative assignment of shifts in the spectra. AFM images were recorded on an NT-MDT AFM microscope using a silicon nitride probe (manufacturer: NanoWorld) with a tip radius of <15 nm, a resonance frequency of 67 kHz, and a force constant of 0.32 N/m. The same probe was used for each sample in tapping mode. Goniometry measurements were conducted using ultrapure water. SE scans were recorded using a J.A. Woollam M-2000 ellipsometer.

### Preparation of H-Terminated Silicon Surface (H–Si(100))

All glassware was washed with 1× Nano-Strip solution (a stabilized formulation of sulfuric acid and hydrogen peroxide), followed by rinsing with water and isopropyl alcohol (IPA) before being dried in an oven overnight at 130 °C. 4 cm^2^ Si(100) substrates were soaked in Nano-Strip at 75 °C for 15 min to produce hydroxy-terminated silicon chips (OH–Si(100)). Following the oxidation, the substrates were immersed in a 5% aqueous hydrofluoric acid (HF) solution for 6 min to chemically etch away the native oxide layer and form hydrogen-terminated silicon. The substrates were then quickly rinsed with water and 2-propanol and dried under filtered nitrogen gas.

### Halogenation of H-Terminated Silicon with *N*-Halogen-succinimides (Hal(H)–Si(100))

Freshly prepared H-terminated Si(100) substrates were placed in a vacuum jar along with 0.5 g of *N*-Hal-succinimide. The jar was evacuated to ∼10^–1^ mbar and heated to a temperature that was 25 °C below the melting point of each *N*-Hal-succinimide molecule (e.g., 150 °C for *N*-bromo-succinimide). These temperatures were selected through experimentation to ensure adequate volatility of the *N*-Hal-succinimide molecules at the vacuum jar pressure (∼10^–1^ mbar). The sample was left in the jar for 2 h to fully vaporize 0.5 g of *N*-Hal-succinimide molecule from the source. Samples were then rinsed with IPA and dried under nitrogen.

### Halogenation of OH-Terminated Silicon with *N*-Halogen-succinimides (Hal(OH)–Si(100))

Freshly prepared H-terminated Si(100) substrates were soaked in Nano-Strip at 75 °C for 15 min, thus undergoing hydroxylation and rendering the surface OH terminated, and placed in a vacuum jar along with 0.5 g of *N*-Hal-succinimide. The jar was evacuated to ∼10^–1^ mbar and heated to a temperature that was 25 °C below the melting point of each *N*-Hal-succinimide molecule for 2 h. Samples were then rinsed with IPA and dried under nitrogen.

### Atomic Layer Deposition of Al_2_O_3_ Thin Films

Deposition of Al_2_O_3_ thin films was carried out using a Cambridge Savannah 200 ALD reactor. Hal-Si(100), H–Si(100), and OH–Si(100) samples were placed in the reactor and heated to 130 °C. Trimethylaluminum (Al(CH_3_)_3_) (Hi-k grade, Air Products) and H_2_O were pulsed for 0.03 and 0.05 s, respectively, by using a nitrogen carrier gas flowing at 20 sccm from sources held at room temperature. The reagent exposures were 6 × 10^–4^ and 1 × 10^–3^ Torr-sec during the Al(CH_3_)_3_ and H_2_O pulses, respectively. The base pressure in the reactor between pulses was 0.3 mbar. A 20 s nitrogen purge followed each precursor pulse. This process was repeated for 20 cycles of deposition.

## Results and Discussion

### Halogenation of H–Si(100) and OH–Si(100) Using *N*-Halogen-succinimides

A mild, vapor-based functionalization method that selectively halogenates H–Si(100) over OH–Si(100) can be applied in AS-ALD processes to (1) create an initial NGS for area-selective thin-film deposition and (2) regenerate a NGS during thin-film deposition to extend the selectivity window. Such selective H–Si(100) halogenation can be achieved by exploring the higher reactivity of the H–Si(100) interfaces. In this study, *N*-Cl/Br/I-succinimides were used to halogenate H–Si(100) under mild vapor-phase conditions. The reactivity of the *N*-Cl/Br/I-succinimides with OH–Si(100) interfaces was also examined to determine the halogenation selectivity between H–Si(100) and OH–Si(100).

**Figure 1:**
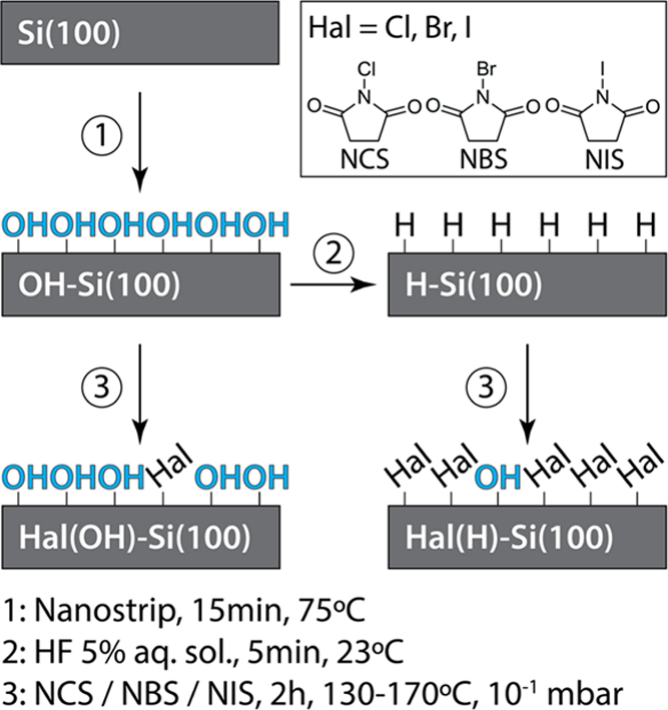
Schematic procedure of reaction steps and process conditions for the vapor-phase halogenation of H–Si(100) and OH–Si(100) with *N*-Hal-succinimides. (1) Si(100) is cleaned and oxidized in Nano-Strip solution before (1 ⇒ 3) direct exposure to an *N*-Cl/Br/I-succinimide molecule in the vapor phase, alternatively (1 ⇒ 2) the new oxide is re-etched in HF and then (2 ⇒ 3) exposed to the *N*-Cl/Br/I-succinimide molecules.

The steps and experimental conditions of these reactions are listed in Figure 1. Si(100) substrates with native oxide were immersed in Nano-Strip solution for 15 min at 65 °C and then rinsed in ultrapure water and isopropanol to remove organic impurities. The native oxide layer was then etched away during a 5 min dip in a 5% HF solution to form a H–Si(100) surface. AFM imaging shown in Figure 1SI confirmed that the HF etch did not affect the surface roughness of the silicon interface. The freshly prepared H–Si(100) surfaces were exposed to an *N*-Cl/Br/I-succinimide molecule for 2 h in a flask evacuated to 10^–1^ mbar and heated to the temperature ∼25 °C below the atmospheric melting point of the succinimide molecules (130–170 °C). Similarly, freshly oxidized OH–Si(100) substrates were also reacted with the *N*-Cl/Br/I-succinimide molecules to assess halogenation selectivity toward H–Si(100) and OH–Si(100). Following the reaction, the substrates were rinsed with isopropanol, dried with filtered nitrogen gas, and analyzed using XPS and contact angle measurements to assess the atomic composition and the halogen coverage before and after the reactions.

**Figure 2:**
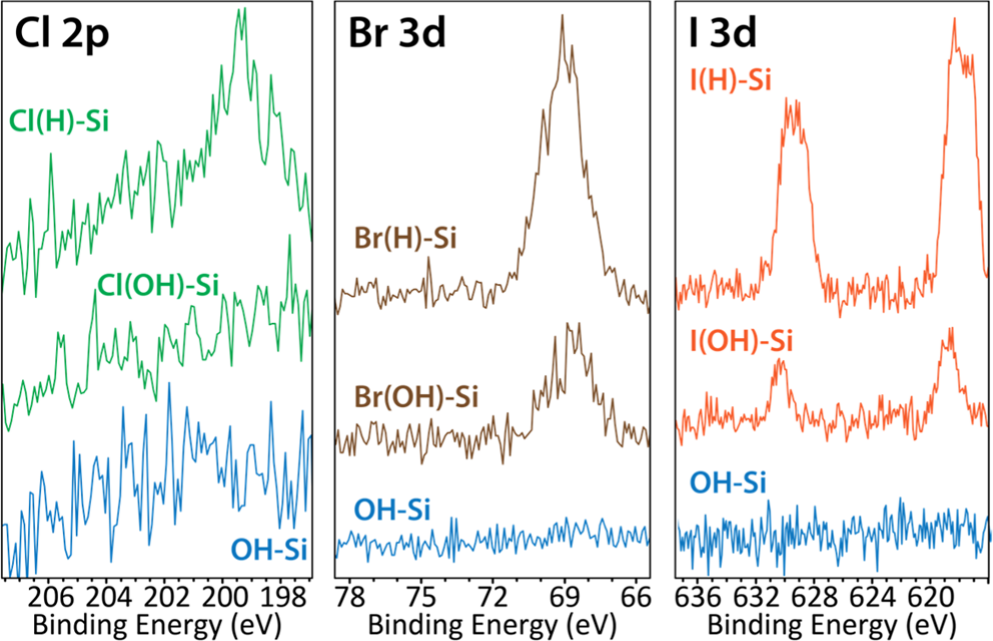
Comparison of XPS spectra from Hal(H)–Si(100) and Hal(OH)–Si(100) surfaces postreaction and an OH–Si(100) standard. Region scans for each respective halogen (Cl 2p, Br 3d, and I 3d) are depicted from left to right.

The XPS Cl 2p, Br 3d, and I 3d spectra in Figure 2 all confirm the formation of Si-Hal bonding on the halogenated H–Si(100) (Hal(H)–Si(100)) substrates. In contrast, the XPS signal intensity on halogenated OH–Si(100) (Hal(OH)–Si(100)) was significantly lower. Table 1 shows the relative XPS peak intensities of Cl 2p, Br 3d, and I 3d electrons adjusted by atomic sensitivity factors (ASFs) and normalized by the total Si 2p peak intensity in each sample. Overall, Table 1 shows that halogenation with *N*-Hal-succinimides resulted in XPS signal intensities 2.8–4 times higher on H–Si(100) than on OH–Si (100). This result was expected due to the more reactive nature of Si–H bonds in interfacial radical-type reactions.^62^

**Table 1:** XPS Ratios of the Hal 2p (or 3d) over Si 2p Electron Signals Were Corrected by the Atomic Sensitivity Factors on Hal(H)–Si(100) and Hal(OH)–Si(100) Substrates after the Halogenation Reaction with *N*-Hal-succinimides^a^

halogenating agents	NCS (Cl)	NBS (Br)	NIS (I)
	XPS signal ratios of Hal 2p (or 3d)/Si 2p electrons		
H–Si(100)	0.028	0.017	0.012
OH–Si(100)	0.010	0.005	0.003
	OH–Si/H–Si halogenation selectivity		
	1:2.8	1:3.5	1:4

a Halogenation selectivity of *N*-Hal-succinimides.

The binding energies of the halogen electrons are consistent with literature data that report halogenation of H–Si(100). Their values suggest the formation of a silicon-halogen species. Specifically, signals at 199.5, 69.5, and 619/631 eV are indicative of Si–Cl, Si–Br, and Si–I formation, respectively.^37,63,64^

Previous studies have relied on XPS to identify the type of halogenated species bonded directly to Si(100). For instance, Silva-Quinones et al. paired XPS measurements with scanning tunneling microscopy (STM) imaging to demonstrate that their method for the chlorination of H–Si(100) resulted in the formation of silicon dichloride (SiCl_2_) surface species, while bromination yielded complete monobromide (SiBr) monolayer formation.^63^ The positions of their Cl 2p and Br 3d peaks are consistent with those in this study and suggest that our method primarily results in the formation of SiCl_2_ and SiBr bonds.

In order to assess the degree of halogen coverage on each surface, we compared theoretical values, derived from the established overlayer model^65^ (eq 1SI), with the values Silva-Quinones et al.^63^ calculated in their study on H–Si(100) halogenation. Their scanning tunneling microscopy (STM) results demonstrated that an overlayer model coverage of 0.4 corresponds to a complete monobrominated Br–Si(100) interface. The overlayer model coverage of the Br(H)–Si(100) substrate in our study was calculated to be 0.35, indicating that the bromination of H–Si(100) with N–Br-succinimide resulted in approximately 88% monobromide termination of the surface-exposed silicon atoms. The surface coverages of the chlorinated and iodinated H–Si(100) substrates were subsequently calculated by comparing the ASF-corrected ratio of Br 3d/Si 2p XPS signals with the ASF-corrected ratios of the Cl 2p/Si 2p and I 3d/Si 2p XPS electron signals (Tables 1 and 2). Our findings suggest that NCS chlorination achieved either an incomplete dichloride termination or a complete mixed mono/dichloride termination of the surface-exposed silicon atoms, while NBS bromination achieved 88% coverage of the monobromide silicon species. However, NIS iodination resulted in only 62% monoiodine coverage of the surface silicon atoms. This decrease in the halogenation efficiency from chlorine to bromine to iodine could potentially be attributed to the increase in the atomic size of the halogen species or to the decrease in the Si-Hal bond energy.

Table 2 shows that the Hal-terminated surfaces display higher hydrophobicity than unreacted OH–Si(100) and similar or lower hydrophobicity than untreated H–Si(100), based on water contact angle measurements of halogenated and nonhalogenated substrates (Figure 2SI). This decrease in hydrophobicity from Cl to I can be attributed to the increasing polarizability of halogen atoms in that order and to partial oxidation of the Si–I interface. The observed water contact angle hysteresis of the halogenated surfaces is likely attributed to the incomplete coverage of the halogen species on the surface and the presence of surface oxide species. As expected, the water contact angles of the halogenated OH–Si(100) substrates are comparable with those of the native OH–Si(100) surface due to the low yield of the halogenation reaction on the oxidized surface.

**Table 2:** Halogen Surface Coverage on Hal(H)-Si(100) Substrates Treated with *N*-Hal-succinimides^a^

halogenating agents	NCS (Cl)	NBS (Br)	NIS (I)
	monohalogen surface coverage (%)		
H–Si(100)	145	88	62
	contact angle (deg) and contact angle hysteresis (deg)		
Hal(H)–Si(100)	72.5 ± 1.6	46.9 ± 0.6	26.2 ± 0.9
	27.1 ± 0.1	24.0 ± 0.1	20.4 ± 0.1
Hal(OH)–Si(100)	21.9 ± 0.4	24.4 ± 0.5	25.2 ± 0.7
	17.9 ± 0.1	19.2 ± 0.1	17.1 ± 0.1
bare OH–Si(100) before halogenation	20.7 ± 0.4		
bare H–Si(100) before halogenation	67.0 ± 0.4		

a Water contact angle measurements of Hal(H)–Si(100) and Hal(OH)–Si(100) substrates and a bare OH–Si(100) substrate before the reaction.

**Figure 3:**
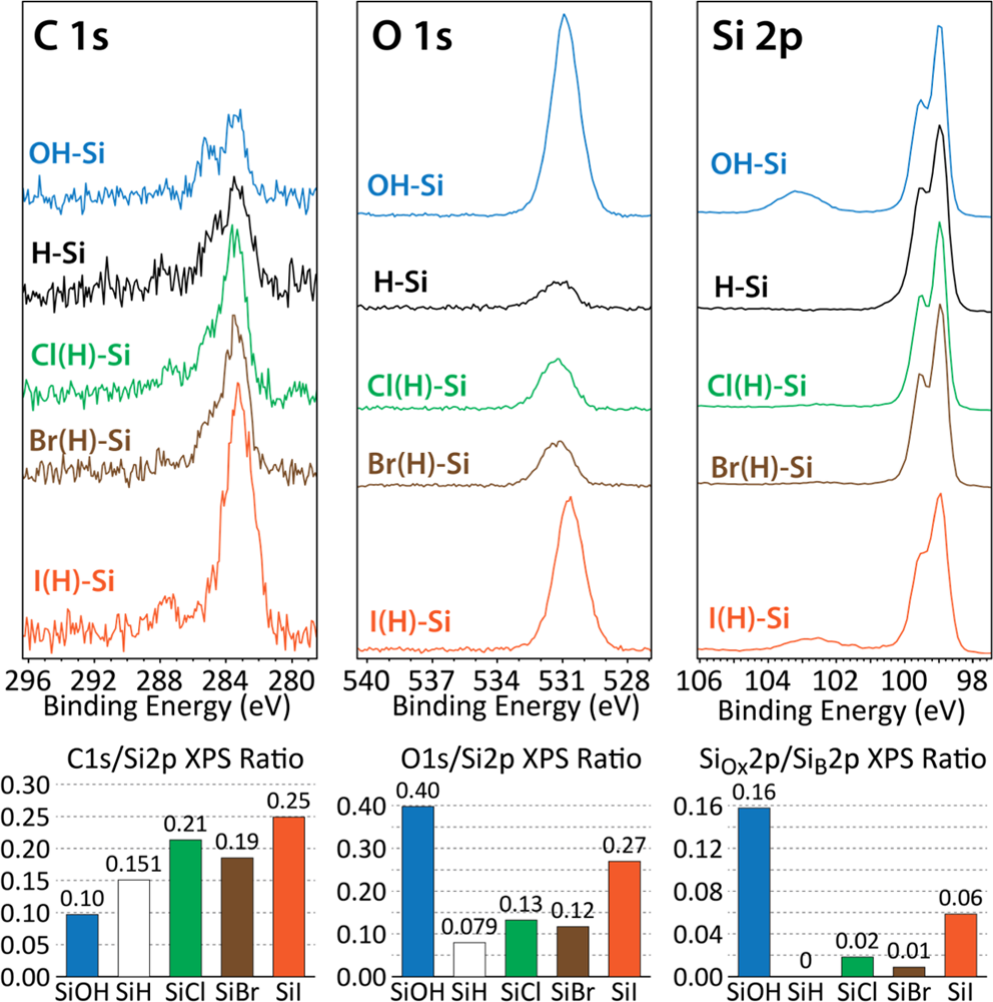
Comparison of XPS region scans of C 1s, O 1s, and SiO_2_ (from Si 2p) spectra depicted from left to right for OH–Si(100), H–Si(100), Cl(H)–Si(100), Br(H)–Si(100), and I(H)–Si(100) surfaces. In the bottom row is histograms showing the quantitative XPS characterization of region scans (C 1s, O 1s, SiO_2_ from Si 2p) for each Hal(H)–Si(100) surface and reference unreacted OH–Si(100) and H–Si(100) surfaces, all normalized by the Si 2p peak intensity which includes contributions from both the bulk Si (Si_B_) and surface oxide (Si_Ox_) interfaces (the values in the Si_Ox_ histogram were normalized by only Si_B_).

XPS region scans of C 1s, O 1s, and SiO_2_ signal (from the Si 2p spectra) were collected to discern compositional differences in the prepared halogenated monolayers (Figure 3). With a negligible SiO_2_ peak observed, and only a slight O 1s and C 1s signal detected, the spectra for the H–Si(100) sample demonstrate that the initial oxidative cleaning and HF etching steps effectively removed a majority of organics from the sample surface before halogenation. The region scans for each of the surfaces posthalogenation show little variability in composition. The primary contribution to each of the C 1s spectra is from C–C/C=C bonds at 284 eV. The highest degree of carbon contamination was seen on I(H)–Si(100), a likely result of the higher polarizability of the iodine atom and therefore the higher surface energy of the entire surface. There is good agreement in the size and shape of the SiO_2_ peaks between both the Cl(H)–Si(100) and Br(H)–Si(100) surfaces and H–Si(100). However, in relation to the OH–Si(100) surface, the SiO_2_ signal for each of the halogenated surfaces is slightly shifted to a lower binding energy. This is most evident when comparing the SiO_2_ peaks for OH–Si(100) and I(H)–Si(100) where there is about a 0.3 eV difference in the peak center. This shift is most likely indicative of the newly formed Si-Hal species found on the halogenated samples and absent on the OH–Si(100) and H–Si(100) references. Overall, the dry halogenation process effectively prevents silicon oxidation for chlorination and bromination. The low O content that is observed on these two surfaces can be attributed to the physisorption of water. The O and SiO_2_ peak areas measured on the I(H)–Si(100) sample were larger likely due to the lower halogen coverage observed on that surface, which allowed surrounding O species to more effectively access the underlying Si–H bonds. This likely explains the higher degree of hydrophilicity that was also observed on I(H)–Si(100). Nevertheless, the degree of Si oxidation that I(H)–Si(100) experiences is still only about a third of what was observed on the reference OH–Si(100) sample.

### Stability of Hal(H)–Si(100) Surfaces in Air

The stability of Cl(H)–Si(100), Br(H)–Si(100), and I(H)–Si(100) in an ambient environment was examined by monitoring the surface composition of each surface via ex situ XPS characterization over a 72 h timespan. After the reaction, each halogenated surface was directly transferred into the XPS for measurements. After collecting the data, the samples were unloaded and kept at ambient laboratory conditions for 4 h. Following this, each sample was reloaded into the XPS for additional testing. This same process was repeated at both 24 and 72 h postreaction.

**Figure 4:**
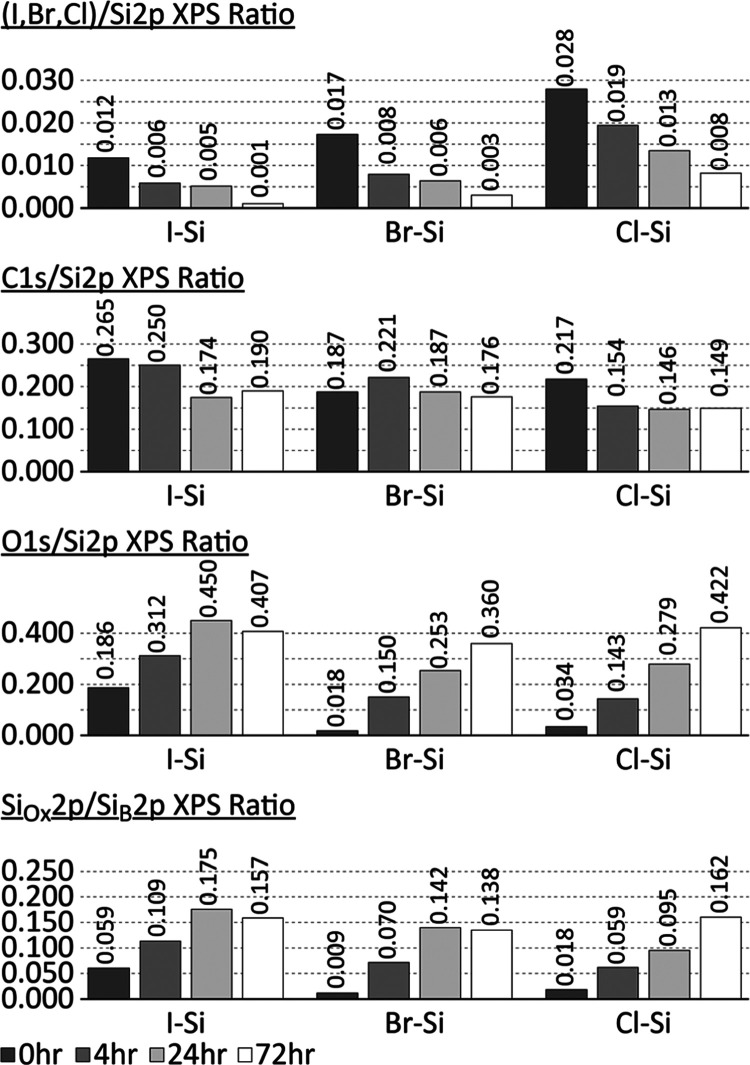
Stability study consisting of histograms showing the quantitative XPS characterization of halogen, C 1s, O 1s, and SiO_2_ (from Si 2p) region scans shown from top to bottom, respectively, for I–Si(100), Br–Si(100), and Cl–Si(100) surfaces shown from left to right, respectively, over a 72 h period of air exposure.

XPS characterization in Figure 4 demonstrated that although each halogenated sample attained roughly the same degree of surface oxidation after 72 h of air exposure, the rate of halogen degradation on Cl(H)–Si(100) was slower than on the other two surfaces. Br(H)–Si(100) and I(H)–Si(100) both experienced more than a 50% drop in halogen signal during the first 4 h, while Cl(H)–Si(100) only exhibited a 32% drop. Deterioration then proceeded slowly during the next 20 h with each surface experiencing less than a 20% additional drop in halogen signal, and by 72 h, there was near-complete desorption of halogen atoms, except on Cl(H)–Si(100) which was retained 29% of its original halogen content. It should be noted that all Cl 2p signals are partially obscured by a Si plasmon, as seen in Figure 3SI, which renders peak identification and characterization slightly more difficult than the other halogen signals. The plasmon was subtracted from all Cl 2p spectra by using a reference Cl 2p spectrum from a bare OH–Si(100) sample.

All three surfaces experienced similar rates of O accumulation in the 24 h postreaction, with I(H)–Si(100) having a head start due to its lower initial halogen coverage, as previously discussed. A reference for a theoretical maximum in Si oxidation is the native oxide layer on the freshly oxidized OH–Si(100) surface in Figure 3, where the Si_Ox_2p/Si_B_2p XPS ratio is 0.16. The SiO_2_ plot in Figure 4 illustrates that I(H)–Si(100) reached the threshold for full oxide growth after 24 h and then leveled off, while Cl(H)–Si(100) reached full growth after 72 h. Only Br(H)–Si(100) exhibited a SiO_2_ signal below the max threshold at the end of 72 h. This surface also demonstrated the lowest O 1s signal after 72 h. Frederick et al. also determined that Br(H)–Si(100) demonstrated the highest resistivity to oxidation in their study where they evaluated the stability of UHV-prepared Cl(H)–Si(100), Br(H)–Si(100), and I(H)–Si(100) in a nitrogen gas environment.^37^ Consequently, after 72 h, the three halogenated surfaces more or less resembled each other with respect to organic and oxide concentration. Within the first 24 h, the results clearly demonstrate that Cl(H)–Si(100) resisted both surface oxidation and halogen deterioration most effectively.

### Hal(H)–Si(100) Surfaces Ability to Inhibit ALD Precursors

Several publications have shown that typical organosilane or phosphonic acid SAMs on Si(100) act as viable ALD resists, but they lose their selectivity due to the hydrolytic desorption promoted by the ALD conditions.^66,67^ Nevertheless, due to the long symmetrical aliphatic chains associated with SAMs, they can provide effective shielding of the underlying surface from the ALD chemistry in the defect-free areas.^21^ Around defects, due to the disruption of the self-assembly, SAMs are less efficient in protecting the substrates from the ALD reactions.^66,68−70^ Such dependence of stability on surface morphology complicates the use of SAM resists on nonideal substrates. Additionally, SAM resists are primarily deposited from solutions (conditions incompatible with in situ resist regeneration).^71,72^ Atomic-scale resists, on the other hand, do not rely on self-assembly and can achieve both higher surface coverage and stronger substrate attachment than SAMs. Potentially, they can also achieve higher resolution due to their smaller size. Certain atomic resists can be deposited in the vapor phase, potentially enabling their in situ deposition within the ALD instrumentation. As halogen monatomic resists can be applied in the vapor phase on a variety of surface geometries, they may represent a more effective alternative to SAMs as ALD inhibitors. Hence, the halogenated monolayers investigated in this study were next examined on whether they (1) formed stable enough bonds with Si(100) to adequately block ALD chemistry and (2) could withstand multiple deposition cycles.

This study is specifically interested in the deposition of aluminum oxide (Al_2_O_3_), the most common metal oxide film deposited via ALD, and is often used in the production of electroluminescent displays and memory capacitors.^31^

**Figure 5:**
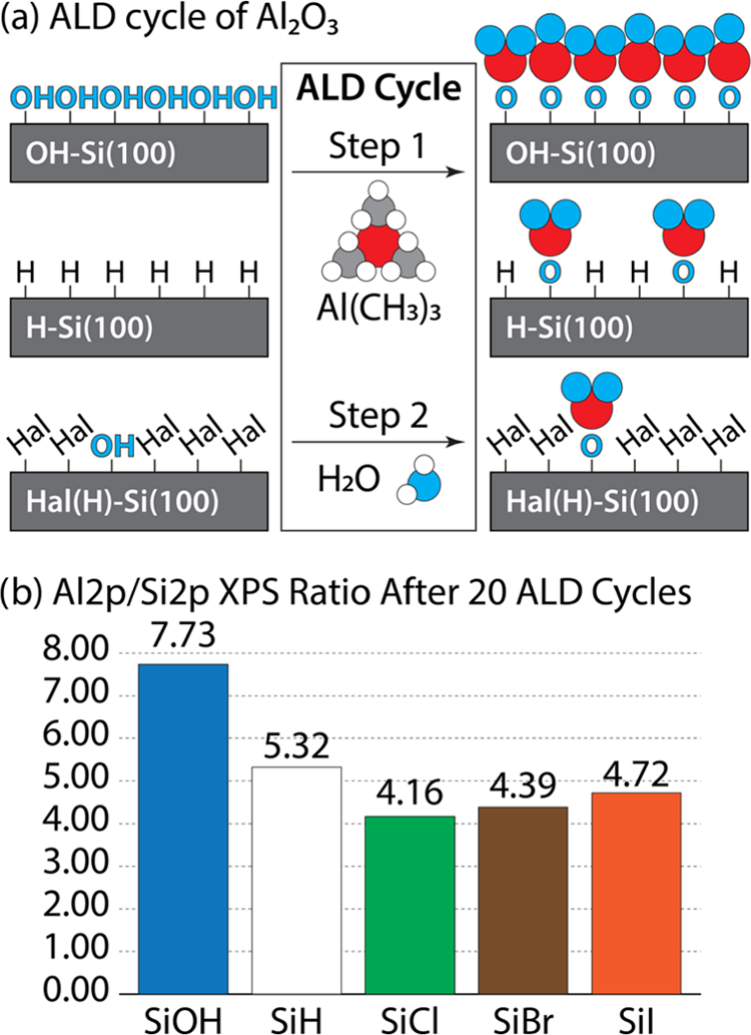
(A) Schematic illustration of the traditional ALD cycle of Al_2_O_3_ onto OH-Si(100), H-Si(100), and Hal(H)-Si(100) surfaces. In step 1, the dosed Al(CH_3_)_3_ precursor readily adsorbs onto the OH-Si(100) surface, while H-Si(100) and Hal(H)-Si(100) surfaces exhibit total and partial blocking of the same molecule, respectively. In step 2, the H_2_O coreactant binds with the adsorbed metal precursor to form a complete metal oxide film on OH-Si(100) and a metal oxide island on Hal(H)-Si(100). Only H-Si(100) retains its original surface, but it must be maintained under high vacuum and temperature. (B) Histogram showing the quantitative XPS characterization of Al 2p region scan for all three halogenated surfaces and reference H-Si(100) and OH-Si(100) surfaces.

The XPS analysis in Figure 5 demonstrates that after 20 cycles of Al_2_O_3_ ALD, the Al-to-Si ratios on H–Si(100) and OH–Si(100) were 5.32 and 7.73, respectively. This illustrates that the ALD chemistry was more selective toward OH–Si(100) which agrees with many studies in the literature.^8,25,31,66,73,74^ For example, Longo et al. used density functional theory (DFT) calculations to denote that the adsorption of the metal oxide precursor (Al(CH_3_)_3_) experiences a higher kinetic barrier on H-terminated surfaces (∼1.5 eV) than on OH-terminated surfaces (∼0.8 eV).^74^ Thus, as OH–Si(100) serves as an ideal GS, it is important to evaluate the ability of each Hal(H)–Si(100) surface to block ALD precursor chemisorption so as to determine their efficacy as potential complementary NGS materials. To test this, Cl(H)–Si(100), Br(H)–Si(100), and I(H)–Si(100) surfaces underwent 20 cycles of Al_2_O_3_ deposition. Figure 5 shows that all three halogenated surfaces exhibited lower Al 2p signals and Al-to-Si composition ratios than those found on a corresponding H–Si(100) surface, thus demonstrating the superior blocking ability of the halogenated surfaces. The Al 2p XPS spectra for each surface are presented in Figure 4SI. Cl(H)–Si(100) was the most effective inhibitor, followed by Br(H)–Si(100) and then I(H)–Si(100). This trend is likely due to the higher halogen coverage on Cl(H)–Si(100) than those on Br(H)–Si(100) and I(H)–Si(100). Interestingly, despite there being a more than 2.5 time difference in water contact angle between the Cl–Si(100) and I–Si(100) surfaces, there was only a 12% difference in their respective Al-to-Si ratios, indicating that atomic polarizability and/or partial surface oxidation do not significantly contribute to the reduction of the ALD selectivity. The ALD growth selectivities reported in Table 3 represent the proportion between the surface concentration of Al atoms on the “inhibiting” NGS relative to that of the OH–Si(100) GS. The selectivities of Cl(H)–Si(100), Br(H)–Si(100), and I(H)–Si(100) were 30.0, 27.6, and 24.2%, respectively. H–Si(100) demonstrated a slightly lower selectivity of 18.5%. Thus, in addition to their potential capability of being intermittently redosed directly into the ALD reactor when selectivity wains (after etching away accumulated NGS Al_2_O_3_/TMA and reforming the halogen-reactive Si–H surface using either a vapor-phase, plasma-based, or atomic layer etching (ALE) method of Si–OH^75−79^), the halogenated substrates also exhibit a higher degree of ALD inhibition than that of H–Si(100).

**Table 3:** Characterization of Al_2_O_3_ Thin Films Deposited onto Sample Surfaces after 20 ALD Cycles Included Thickness Calculations from ARXPS and XPS Measurements and AFM Roughness Measurements^a^

surface	thickness (nm) (based on XPS)	ALD growth rate (nm/cycle)	selectivity (%) (based on XPS)	AFM RMS roughness (nm)
OH–Si(100)	2.16	0.11	--	0.49
H-Si(100)	1.49	0.07	18.5	0.53
Cl(H)–Si(100)	1.16	0.06	30.0	0.21
Br(H)–Si(100)	1.23	0.06	27.6	0.76
I(H)–Si(100)	1.32	0.07	24.2	0.78

a Selectivity is defined as a proportion between the surface concentration of Al atoms on the OH–Si(100) reference and the surface concentration of Al atoms on the Hal-Si(100) surfaces, all values found using XPS and normalized to the Si 2p signal.


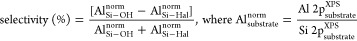


**Figure 6:**
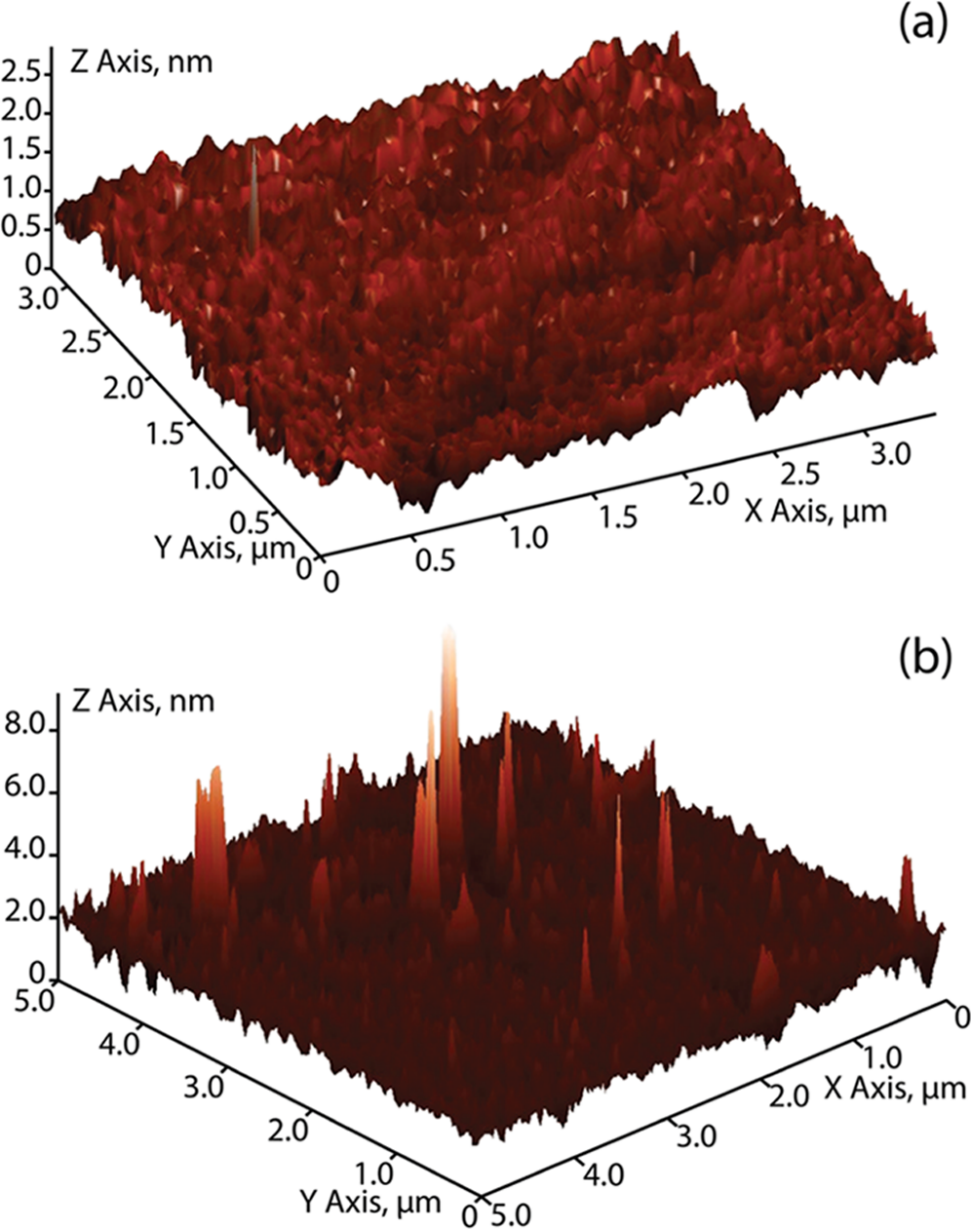
Three-dimensional AFM roughness topographies taken for the (a) OH–Si(100) and (b) Br(H)–Si(100) surfaces post ALD.

The inhibiting character of the halogenated surfaces can also be defined by the degree of conformation of the deposited thin films. For example, in Figure 6, three-dimensional AFM roughness topographies are displayed for the OH–Si(100) and Br(H)–Si(100) surfaces post ALD. The OH–Si(100) surface exhibits both a lower RMS roughness measurement and a smoother topography compared to that of the Br(H)–Si(100) surface. This is as expected since the brominated monolayer does not promote homogeneous nucleation of the precursor molecules and thus the gradual Al_2_O_3_ film growth should remain uneven. And this phenomenon is observed in the surface topography for the Br(H)–Si(100) surface where there is a greater number of defects scattered across the entire area of measurement, whereas OH–Si(100) exhibits a more homogeneous surface.

Angle-resolved XPS (ARXPS) measurements were used to calculate the thickness of the deposited Al_2_O_3_ film on an OH–Si(100) GS.^80−84^ The ARXPS measurements were inputted into eq 2SI, which describes the relationship between the XPS area intensity of the substrate electrons, the electron collection angle, and the thickness of the film that covers the substrate. The values for the mean free path of Si 2p electrons in the SiO_2_ and Al_2_O_3_ layers were calculated using the computational NIST model. The ARXPS thickness for the surface layer system comprising both a native SiO_2_ base layer and the top Al_2_O_3_ ALD film was calculated to be 4.26 nm. A ellipsometry measurement for the native SiO_2_ base layer shown in Figure 6SI demonstrates that the thickness of this layer is approximately 2.10 nm. Thus, the ARXPS measured thickness of the Al_2_O_3_ film on an OH–Si(100) after 20 cycles was determined to be 2.16 nm, as reported in Table 3.

The Al_2_O_3_ film thickness on the OH–Si(100) surface was used to correlate measured Al 2p/Si 2p XPS peak ratios on Si-OH, Si-H, and Si-Hal substrates with the thicknesses of the deposited alumina layers on these substrates (ARXPS method was not applied due to the uncertainty of how the ALD chemistry alters the thickness of the underlying halogen sublayers). The resulting film thicknesses on the NGS are reported in Table 3, which shows that the thinnest Al_2_O_3_ film was observed on Cl(H)–Si(100), followed by Br(H)–Si(100) and then I(H)–Si(100). This trend in film thickness mirrors that of the XPS halogen-to-silicon signal ratios, halogen coverage values, water contact angle, and blocking ability determined from XPS for all three surfaces. All three halogenated surfaces exhibited ALD film thicknesses lower than H-Si(100). Despite the three halogenated surfaces exhibiting similar Al-to-Si XPS ratios, when accounting for a higher 24 h air stability observed on Cl-Si(100), this material appears to be a more suitable candidate for Al_2_O_3_ ALD surface blocking. Overall, the reduced reactivity that each halogenated surface showed toward the ALD chemistry demonstrates that they are each suitable candidates for effective ALD resists and when paired with an ALD growth material, such as OH–Si(100), can participate in chemoselective processing schemes such as AS-ALD.

## Conclusions

In this study, XPS, ellipsometry, and contact angle goniometry measurements were used to demonstrate the covalent bonding of halogenated monolayers to a H-Si(100) surface prepared via a dry reaction process. NCS, NBS, and NIS were all found to be effective halogenating agents of H-Si(100). However, chlorination resulted in the highest halogen surface coverage by a significant margin, followed by bromination and then iodination. This approach exhibited exclusive bonding of halogen atoms to silicon while maintaining a low rate of oxidation of the underlying silicon interface, especially for chlorination and bromination. Stability tests in air were then undertaken to determine how long each surface could resist degradation and oxidation. Over the course of 24 h, Cl(H)–Si(100) demonstrated the strongest resistance to both halogen deterioration and SiO_2_ growth, followed by Br(H)–Si(100) and then I(H)–Si(100). At the end of 72 h, each surface more or less resembled each other with respect to organic and oxide concentration; however, Cl(H)–Si(100) was able to retain about a quarter of its original halogen coverage, something the other two surfaces did not achieve. Surfaces halogenated via a vapor-phase reaction can more feasibly be implemented into vacuum-based processes such as ALD. In such a process, the halogenating molecule can be dosed into the reactor chamber in the same manner as the ALD chemistry. Applying inhibiting molecules to a NGS on a sample surface will allow for patterned deposition of the desired thin film, using atoms as building blocks for synthesizing materials from the bottom-up. As a proof of principle, surfaces halogenated following our enumerated protocol underwent traditional ALD in order to examine the newly formed surface’s blocking ability against a metal oxide precursor. Consequently, SE and AFM data demonstrated the improved shielding ability of the halogenated monolayers relative to those of H-Si(100) and OH–Si(100). Once again, Cl(H)–Si(100) demonstrated the most effective blocking ability against the ALD chemistry, followed by bromination and then iodination. However, the reduced reactivity that each halogenated surface showed with the ALD chemistry demonstrates that they are each suitable candidates for effective ALD resists and when paired with an ALD growth material, such as OH–Si(100), can participate in chemoselective processing schemes such as AS-ALD.

## Supporting Information Available

AFM images of the Si-H and Si-OH substrates, Haber and Lewis’s substrate-overlayer model equation, water contact angle measurement of the halogenated substrates, XPS spectra of all substrates, ARXPS Al_2_O_3_ film thickness measurement on the Si-OH substrate, and ellipsometry topographies of the Si-OH substrate (PDF)

## Abbreviations

(AS-ALD): area-selective atomic layer deposition
(GS): growth surface
(NGS): nongrowth surface
(H-Si(100)): hydrogen-terminated silicon
(OH–Si(100)): hydroxyl-terminated silicon
(UHV): ultrahigh vacuum
(Hal-Si(100)): halogenated Si(100)
(Cl-Si(100)): Cl-terminated Si(100) surface
(Br-Si(100)): Br-terminated surface
(H-SiQDs): H-terminated silicon quantum dots
(NCS): *N*-chlorosuccinimide
(NBS): *N*-bromosuccinimide
(NIS): *N*-iodosuccinimide
(XPS): X-ray photoelectron spectroscopy
(ARXPS): angle-resolved X-ray photoelectron spectroscopy
(SE): spectroscopic ellipsometry
(SEM): secondary electron microscopy
(STM): scanning tunneling microscopy
(AFM): atomic force microscopy
(BE): binding energy
(SAMs): self-assembled monolayers
(DFT): density functional theory
(ASF): atomic sensitivity factors
