# Supplementary material for: Vapor-Phase Halogenation of Hydrogen-Terminated Silicon(100) Using N-Halogen-succinimides
Source: ACS Appl Mater Interfaces. 2023 Nov 15;15(47):55139–49. doi: 10.1021/acsami.3c13269 (PMC10694808; doi:10.1021/acsami.3c13269)
Supplement: Supplementary file 1 — am3c13269_si_001.pdf [file am3c13269_si_001.pdf]

Supporting Information

Vapor-Phase Halogenation of Hydrogen-Terminated Silicon(100) Using N-Halogen-Succinimides

Patrick R. Raffaelle[†], *George T. Wang*[‡], *Alexander A. Shestopalov*[†*]

[†] *Department of Chemical Engineering, Hajim School of Engineering and Applied Sciences, University of Rochester, Rochester, New York 14627, United States.*

[‡] *Sandia National Lab, United States.*

[*]*Corresponding Author. E-mail:* alexander.shestopalov@rochester.edu

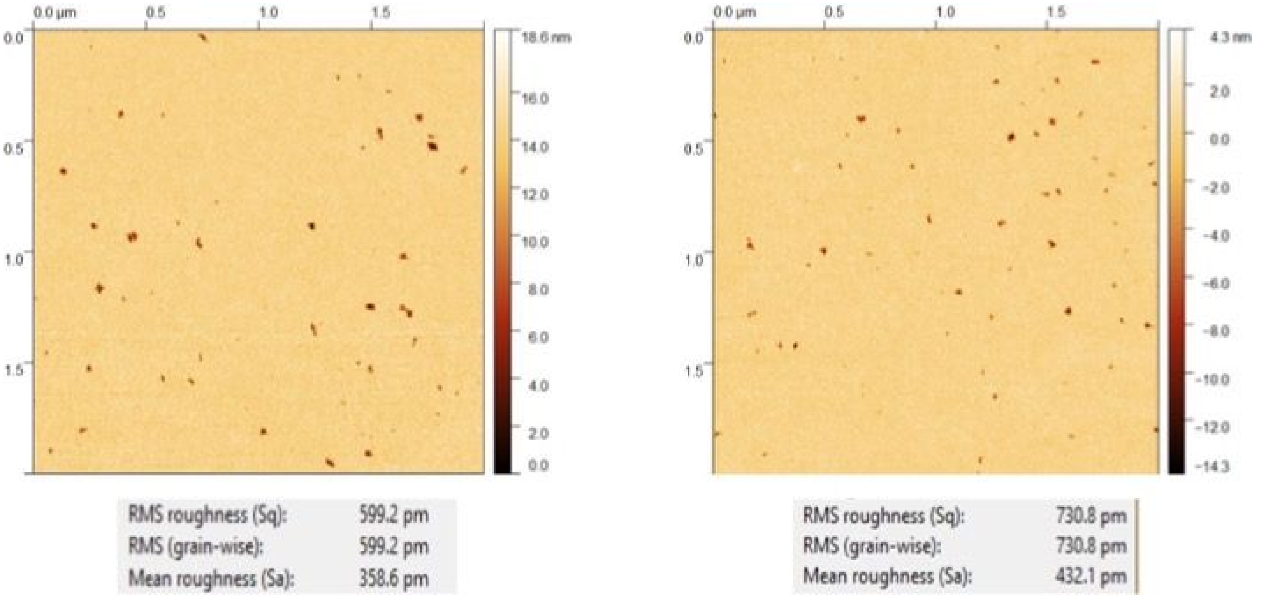

Figure 1SI. AFM scans of (Left) bare OH-Si(100) surface and (Right) H-Si(100) etched with 5% HF. Statistical analysis posted below each image demonstrates that etching of the native SiO_2 layer only resulted in a 131.6 pm increase in RMS surface roughness. Visually, the two images appear similar despite a 10.0+ nm reduction in nominal surface thickness due to removal of the SiO_2 layer. Additionally, there does not appear to be a significant increase in pitting on the HF-treated surface.

Haber and Lewis's substrate-overlayer model (Φ) was used to calculate surface coverage of each halogen monolayer. This model is based on the assumption that the newly formed halogen species comprise a single monolayer that interfaces directly with the silicon substrate. All other organics are assumed to sit on top of this halogen layer.

$$\Phi_{Hal} = \left[\frac{\lambda \sin\theta}{a_{Hal}}\right]\left(\frac{ASF_{Si}}{ASF_{Hal}}\right)\left(\frac{\rho_{Si}}{\rho_{Hal}}\right)\left(\frac{I_{Hal}}{I_{Si}}\right)$$

Equation 1SI Where λ is the penetration depth (calculated for each element using escape depth); θ is the angle in which electrons take off from sample surface (90.0°); a_{Hal} is the atomic diameter of the halogen species; ASF is the atomic sensitivity factor used to scale the measured XPS peak areas so that variations in peak size are representative of the amount of material on the sample surface; ρ is the vapor density of each element; and I is the integrated area under the XPS peaks, processed by CasaXPS software.

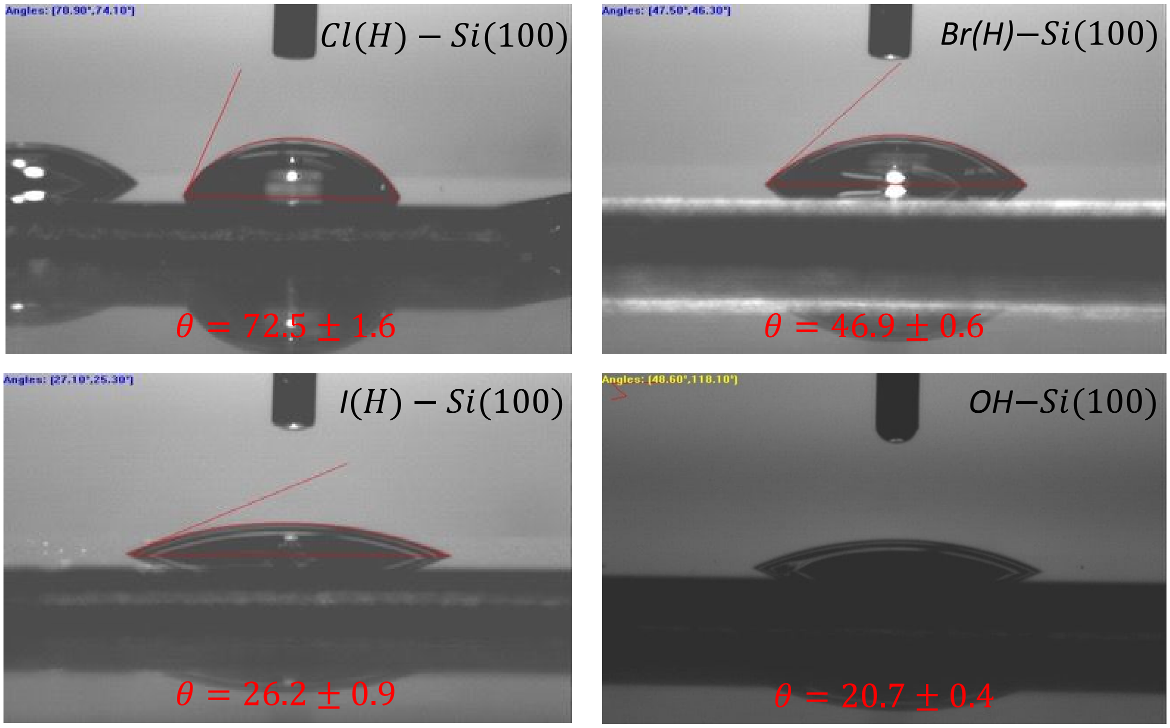

Figure 2SI. Water contact angle pictures of the three halogenated surfaces and a reference OH-Si(100) surface. Surface hydrophobicity rises with increasing halogen coverage.

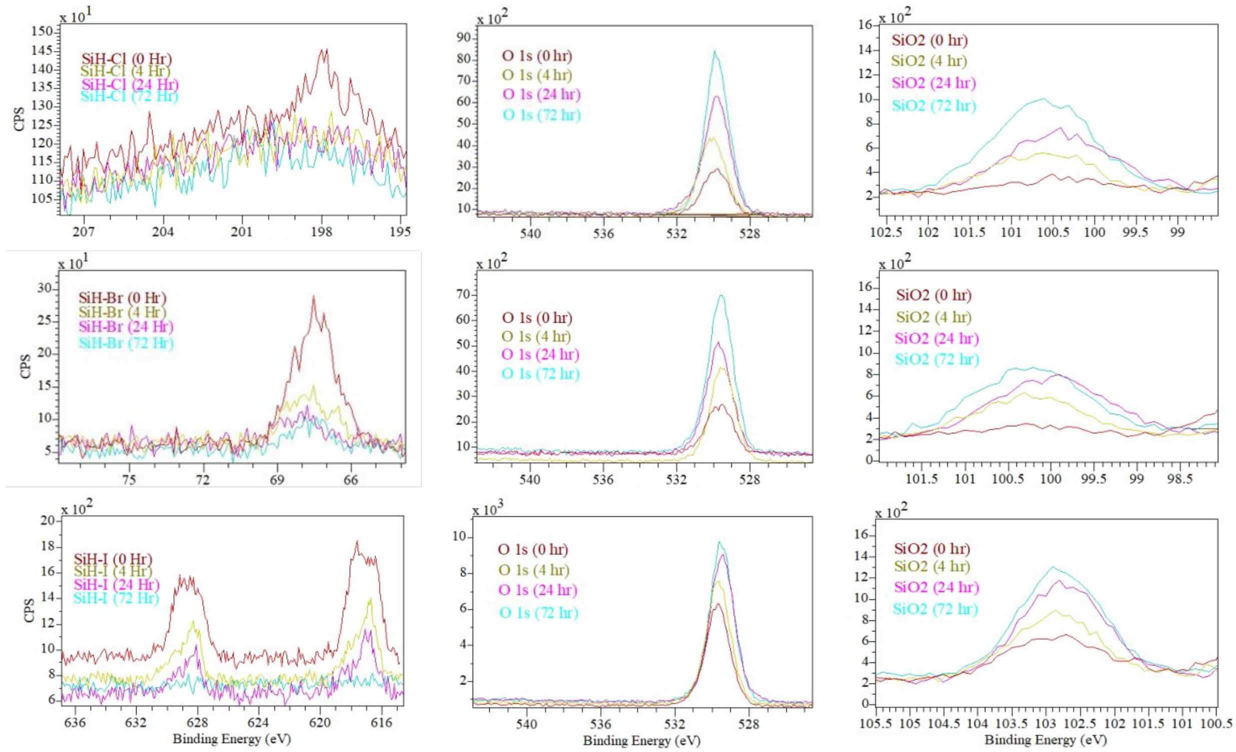

Figure 3SI. XPS spectra showing changes in Hal(H)-Si(100)composition over a 72 hour period. Region scans for each respective halogen (Cl 2p, Br 3d, I 3d) are depicted in row 1, panels A-C. The O 1s spectra and SiO2 signal from the Si 2p spectra for each sample set are depicted in rows 2 and 3, respectively. Scans of halogenated substrates after 0 hr, 4 hr, 24 hr, and 72 hr of air exposure are represented by maroon, olive, pink, and turquois lines, respectively.

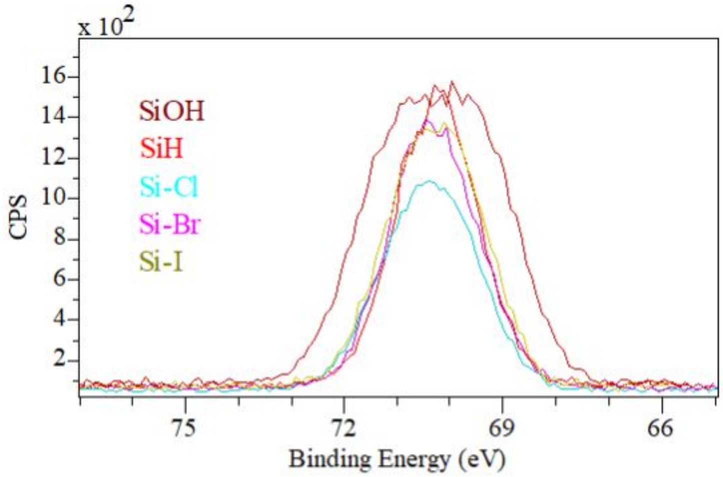

Figure 4SI. Al 2p XPS spectra for Cl(H)-Si(100), Br(H)-Si(100), I(H)-Si(100), H-Si(100), and OH-Si(100) each represented by the maroon, olive, pink, turquois, and red lines, respectively.

ARXPS was used to collect background Si 2p signals at varying take-off angles and fitted into **Equation 2SI** to determine the total thickness of the film layers on top of the bulk silicon interface based on the intensity of Si 2p electrons detected through the layer system (namely SiO_2 and Al_2O_3 layers). The XPS spectra were taken from an extended analytical area (~600 × 900 µm) to maintain an adequate signal-to-noise ratio and to limit the molecular system's exposure to the X-ray radiation. Because of this wide analytical area, a drop in the Si 2p signal intensity was observed at the higher takeoff angles associated with the shallow depth of field of the XPS analyzer. The correction factors for this drop in intensity were measured using a bare Si substrate, in which both SiO_2 and Al_2O_3 layers were absent, thus the change in the substrate intensity at different collection angles is not related to the attenuation of the substrate Si 2p electrons by the ALD film material and is only associated with the change in the sample focal area. This correction factor was then used to adjust the signal intensities of the Si 2p scans from the corresponding OH-SI(100) substrate with 20 cycles of Al_2O_3 deposited on them.

$$I_{Si(s)corr} = A(\theta)_{corr} \times I_{Si(s)} = I_{Si(s)}^{inf} \times exp\left[-\frac{d}{\lambda_{Si(m)} \times \cos(\theta)}\right]$$

Equation 2SI where $I_{Si(s)corr}$ is the corrected area intensity of silicon in the substrate; $A(\theta)_{corr}$ is the correction factor measured on a bare Si substrate; $I_{Si(s)}$ is the measured area intensity of Si in the substrate; $I_{Si(s)}^{inf}$ is the bulk area intensity of Si in the unfunctionalized substrate; $\lambda_{Si(m)}$ is the inelastic mean free path (the attenuated length) of Si 2p electrons in the monolayer; d the monolayer thickness; and θ the collection angle between the sample normal and the analyzer. The corresponding signal intensities, correction factors, and collection angles are reported in **Table 1S**.

Table 1S. Si 2p ARXPS substrate peak areas, collection angles and correction factors used in the **Equation 2SI** thickness calculations of the Al_2O_3 film deposited onto OH-Si(100) after 20 ALD cycles.

Collection Angle (θ)	1/Cos(θ)	I_{Si2p}	Correction Factor $A(\theta)_{corr}$	$I_{Si(s)corr} = A(\theta)_{corr} \times I_{Si(s)}$	ln ($I_{Si(s)corr}$)
0	1	7936.44	1	7936.44	8.98
15	1.0353	6466.11	1.034	6682.72	8.81
30	1.1547	5489.22	1.150	6310.96	8.75
45	1.4142	3290.14	1.452	4777.28	8.47
60	2	824.76	2.600	2144.71	7.67

Using these parameters, **Figure 5SI was** generated and demonstrated a strong linear fit. According to **Equation 2SI**, we derived total surface layers thickness from the slope of the profile.

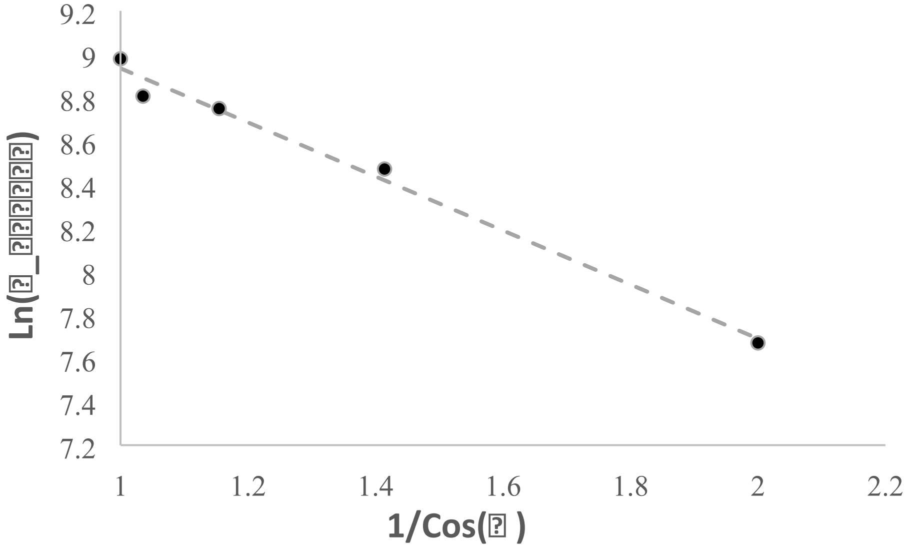

Figure 5SI. ARXPS Experiments of the SiO_2 and Al_2O_3 layers on OH-Si(100) following 20 ALD cycles: Linear Fit of the Corrected Si 2p Signal Areas from the Si Substrate as a Function of 1/cos(θ),

The measured thicknesses (**Figure 5SI**) of the layers was 42.563Å or 4.2563 nm. And after accounting for the thickness of just SiO_2 on a OH-Si(100) reference, nominally set at 2.10 nm based on a previous ellipsometry measurement shown in **Figure 6SI**, the thickness of the Al_2O_3 film deposited on OH-Si(100) after 20 ALD cycles was calculated to be 2.16 nm.

SiO2 Ellipsometry thickness measurement on OH-Si(100) substrate

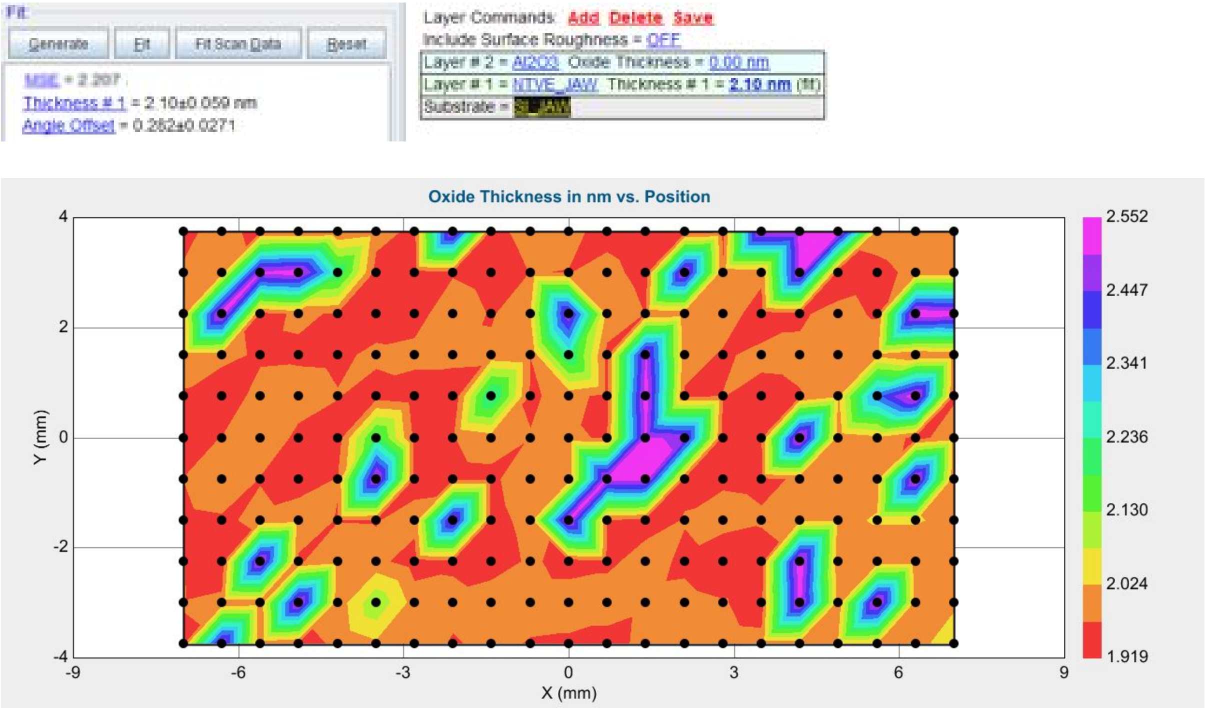

Figure 6SI. Spectroscopic ellipsometry measurement of a reference OH-Si(100) sample following rinsing. The average thickness across a 8 x 14 mm surface was determined to be 2.10 $\pm$ 0.059 nm.
